# Conflicts of Interest Among Authors of Systematic Reviews and Meta-analyses Investigating Interventions for Melanoma: Cross-sectional Literature Study

**DOI:** 10.2196/25858

**Published:** 2021-06-07

**Authors:** Zane Rulon, Kalyn Powers, J Michael Anderson, Michael Weaver, Austin Johnson, Micah Hartwell, Matt Vassar

**Affiliations:** 1 Center for Health Sciences Oklahoma State University Tulsa, OK United States; 2 College of Pharmacy Southwestern Oklahoma State University Weatherford, OK United States; 3 College of Osteopathic Medicine Kansas City University of Medicine and Biosciences Joplin, OK United States

**Keywords:** conflicts of interest, industry sponsorship, melanoma, cross-sectional analysis, systematic review, meta-analysis

## Abstract

**Background:**

Previous studies have highlighted the potential influence that industry relationships may have on the outcomes of medical research.

**Objective:**

We aimed to determine the prevalence of author conflicts of interest (COIs) in systematic reviews focusing on melanoma interventions, as well as to determine whether the presence of these COIs were associated with an increased likelihood of reporting favorable results and conclusions.

**Methods:**

This cross-sectional study included systematic reviews with or without meta-analyses focusing on interventions for melanoma. We searched MEDLINE and Embase for eligible systematic reviews published between September 1, 2016, and June 2, 2020. COI disclosures were cross-referenced with information from the CMS (Centers for Medicare & Medicaid Services) Open Payments database, Dollars for Profs, Google Patents, the United States Patent and Trademark Office, and previously published COI disclosure statements. Results were quantified using descriptive statistics, and relationships were evaluated by Fisher exact tests.

**Results:**

Of the 23 systematic reviews included in our sample, 12 (52%) had at least one author with a COI. Of these 12 reviews, 7 (58%) reported narrative results favoring the treatment group and 9 (75%) reported conclusions favoring the treatment group. Of the 11 systematic reviews without a conflicted author, 4 (36%) reported results favoring the treatment group and 5 (45%) reported conclusions favoring the treatment group. We found no significant association between the presence of author COIs and the favorability of results (*P*=.53) or conclusions (*P*=.15).

**Conclusions:**

Author COIs did not appear to influence the outcomes of systematic reviews regarding melanoma interventions. Clinicians and other readers of dermatology literature should be cognizant of the influence that industry may have on the nature of reported outcomes, including those from systematic reviews and meta-analyses.

## Introduction

According to the US Centers for Disease Control and Prevention [1], there were over 77,000 new cases of melanoma annually between 2012 and 2016, with an incidence rate of 21.8 per 100,000. During the same period, 9000 individuals died from melanoma each year. The estimated annual cost of melanoma treatment in the United States for people over 65 years old was estimated to be US $390 million in 2010 [1]. Due to the prevalence of melanoma and the cost of treatment, improved treatment strategies and novel interventions are critically needed. Well-conducted systematic reviews—considered the highest level of evidence (level 1a) [2]—are routinely used for developing guidelines, assessing novel treatments, and informing clinical decision making [3]. The two most recent clinical practice guidelines from the American Society of Clinical Oncology—Systemic Therapy for Melanoma and Sentinel Lymph Node Biopsy for Melanoma—both include systematic reviews to support their recommendations [4,5]. These guidelines influence physician decision making and patient care. Any bias in the systematic reviews can affect the validity of the data presented.

When appraising results of systematic reviews, it is important to consider whether the authors have industry ties or other conflicts of interest (COIs), as these competing interests may introduce bias that can have downstream effects on patient care [6]. The field of dermatology is not exempt from potential bias from industry ties. For example, dermatologists received more than US $34 million in industry payments in 2014 [7]. Further, Feng et al [8] reported that 73% of all dermatologists accepted industry payments. Considering the nonnegligible presence of industry in the field of dermatology, efforts to increase the transparency of these clinician-industry relationships have been made in hopes of mitigating industry bias within the field.

With the goal of minimizing potential bias, the Physician Payments Sunshine Act was passed in 2010, which requires all physicians to publicly disclose their corroboration with pharmaceutical and medical industries [9]. Since then, further improvements have been made to induce disclosures of industry ties. One such improvement was the creation of the publicly accessible CMS (Centers for Medicare & Medicaid Services) Open Payments database [7], which catalogs all financial relationships between US physicians and industry. According to a study by Young et al [10], nearly two-thirds of people surveyed rated transparency as somewhat or very important; however, nearly 90% of the same subjects had never heard of the CMS Open Payments database. These findings demonstrate that this tool is grossly underutilized by the patient population. With access to these records, Tringale et al [11] found that 48% of physicians accepted payments, totaling US $2.4 billion in one year. Further investigations have found that these relationships among US-based physicians and industry may act as a nidus for COIs [12-16].

While recognition of these relationships is increasing, little literature exists on the pervasiveness of financial and nonfinancial COIs among authors of systematic reviews and meta-analyses [17-19]. While financial relationships are often considered the most influential contributors to possible COIs, other potential conflicts may arise from personal, academic, and intellectual factors [20]. Any COIs among authors of systematic reviews regarding melanoma treatments have the potential to affect patient care and, thus, warrant evaluation [21-23].

To our knowledge, no study has assessed COIs among this group of authors. In this analysis, we strive to identify the nature and types of COIs, both disclosed and undisclosed, of authors of systematic reviews on melanoma therapies. Additionally, we aim to evaluate whether an association exists between sponsorship of systematic reviews and the results and conclusions reported.

## Methods

### Overview

To enhance the transparency and reproducibility of our work, we have provided our study materials, methods, and protocol on Open Science Framework [24]. While drafting this manuscript, we followed the PRISMA (Preferred Reporting Items for Systematic Reviews and Meta-Analyses) guidelines [25] and Murad and Wang’s guidelines for reporting meta-epidemiological methodology [26].

### Search Strategy

We searched MEDLINE (Ovid) and Embase (Ovid) on June 2, 2020, for systematic reviews with or without meta-analyses specific to the treatment of melanoma. Our exact search string can be found in Multimedia Appendix 1.

### Screening

Two authors (ZR and KP) screened search returns by title and abstract in a duplicate, masked manner. After title and abstract screening, full texts were screened according to the eligibility criteria described below. Discrepancies were resolved by a group consensus meeting, with third-party adjudication, if necessary.

### Eligibility Criteria

To be included, articles must have (1) been considered a systematic review or meta-analysis according to the PRISMA-P (PRISMA for Protocols) definition [27]; (2) been a head-to-head comparison of a specific intervention, or combination of interventions, to another intervention or to a placebo or standard of care; (3) been specific to the treatment of melanoma; (4) been published between the dates of September 1, 2016, and June 2, 2020; (5) been published in English; and (6) synthesized data from human participants. The dates of inclusion were based on the International Committee of Medical Journal Editors (ICMJE) recommendation, which states that authors should disclose COIs that occurred 36 months prior to journal submission [28]. By including systematic reviews published after September 1, 2016, we were able to cross-reference reported payments on the CMS Open Payments database—which went live in September 2013—in the 36 months prior to the dates of publication of the systematic reviews within our sample to ensure compliance with ICMJE’s COI disclosure policy.

### Training

Before the study began, investigators received an online training overview. Training included details regarding the study design, objectives, protocol, materials, and data extraction from one systematic review as an example. This training session is available online for reference [24].

### Data Extraction

The same investigators (ZR and KP) who performed study screening also completed data extraction in a masked, duplicate fashion using a pilot-tested Google Form. The full texts of the included studies were analyzed for general study characteristics, including the following: (1) PubMed identification number and/or DOI (Digital Object Identifier), (2) name of journal, (3) date of publication, (4) author names, (5) treatment interventions being compared, (6) affiliations for the first and last authors, (7) funding source, (8) complete COI statement, (9) whether the systematic review or meta-analysis addressed risk of bias (RoB), (10) the verbatim RoB statement, (11) whether a systematic review author was also an author on one or more of the primary studies included in the review (yes or no), (12) the total number of self-cited primary studies, (13) whether an overall pooled effect estimate was calculated (yes or no), (14) the statistical significance of the pooled effect estimate, and (15) whether narrative results and conclusions favored the treatment or comparison group (eg, placebo, standard of care, or control). For the purpose of our study, *conclusion* was defined as the combined discussion and conclusion sections of the review.

### Favorability of Narrative Results and Conclusions

Narrative results and conclusions were deemed as *favorable*, *unfavorable*, or *mixed or inconclusive*. While appraising the results section, *favorable* was assigned when only positive results were reported. *Unfavorable* was assigned when only negative results were reported. *Mixed or inconclusive* was assigned if both positive and negative results were reported. While appraising the conclusion, *favorable* was assigned when authors stated or implied favorability of the intervention group over the comparator group. *Unfavorable* was assigned when authors stated or implied favorability of the comparator group over the intervention group. *Mixed or inconclusive* was assigned if the conclusion section did not meet criteria for *favorable* or *unfavorable* (eg, reporting negative population outcome but positive subgroup analysis).

### Identification of Undisclosed COIs

**Figure 1:**
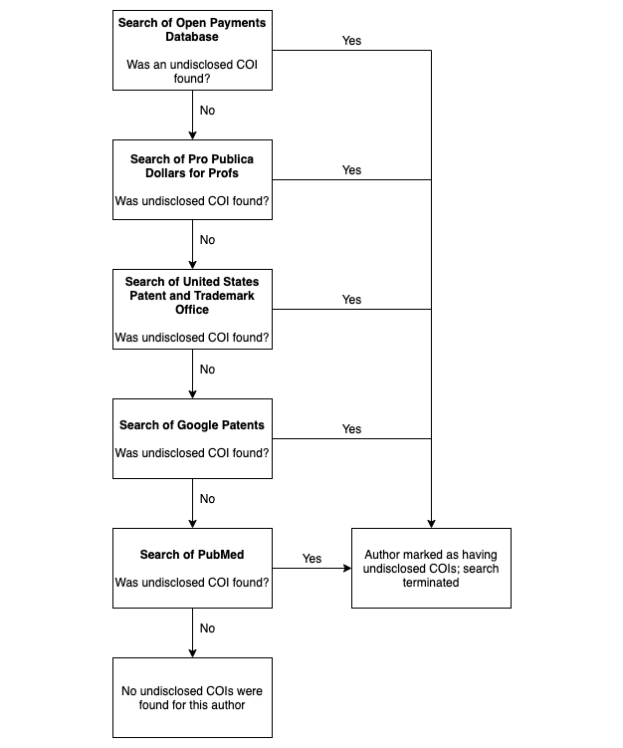
Stepwise search for undisclosed conflicts of interest (COIs) among systematic review authors.

Our search for undisclosed COIs was undertaken using the stepwise strategy outlined in Figure 1. We used a similar search strategy used by Mandrioli et al [29], with slight modifications. These modifications included the use of additional databases: the CMS Open Payments database, Dollars for Profs, and the United States Patent and Trademark Office (USPTO). Multimedia Appendix 2 describes each database. All authors were searched for within these databases for undisclosed COIs, regardless of disclosure status. To ensure the accuracy of data collection, MW used the Python programming language (Python Software Foundation) to create database-specific search strings for the USPTO, Google Patents, and PubMed. If results from the patent searches could not be definitively linked to the author in question, we erred on the side of caution and did not consider this as an undisclosed COI. Based on recommendations for COI disclosure offered by the ICMJE, we limited our search of PubMed to include studies published in the 36 months prior to the date of the systematic review included in our sample. Author COI disclosure statements from the PubMed search results were cross-referenced with the COI disclosure statement found in the systematic review from our sample to determine if previously published studies included additional COIs not disclosed in the systematic review from our sample. In the event that more than 10 records were returned from our PubMed search, each investigator (ZR and KP) individually assigned random numbers to the records and screened the first 10 randomized records for an undisclosed COI. This stepwise search process was continued until an undisclosed COI was discovered, at which point the search was terminated and the author was considered to have an undisclosed COI [29].

### RoB Evaluation

We used the Cochrane Collaboration’s criteria to assess the risk of funding bias in the included systematic reviews, as well as the four items used by Mandrioli et al [29]. Overall RoB was determined using the following criteria: (1) selection for inclusions and exclusions was explicit and *well-defined* and could be replicated by others, (2) the study inclusion method involved two or more assessors selecting studies, (3) search strategies were comprehensive, and (4) studies controlled for methodological differences that may introduce bias. We considered the RoB to be high if fewer than three items received a satisfactory *yes* answer.

### Statistical Analysis

Results were calculated and reported as descriptive statistics. Relationships between systematic review characteristics and outcomes were evaluated by Fisher exact tests, when possible. Stata 16.1 (StataCorp, LLC) was used for all analyses.

## Results

### Overview

**Figure 2:**
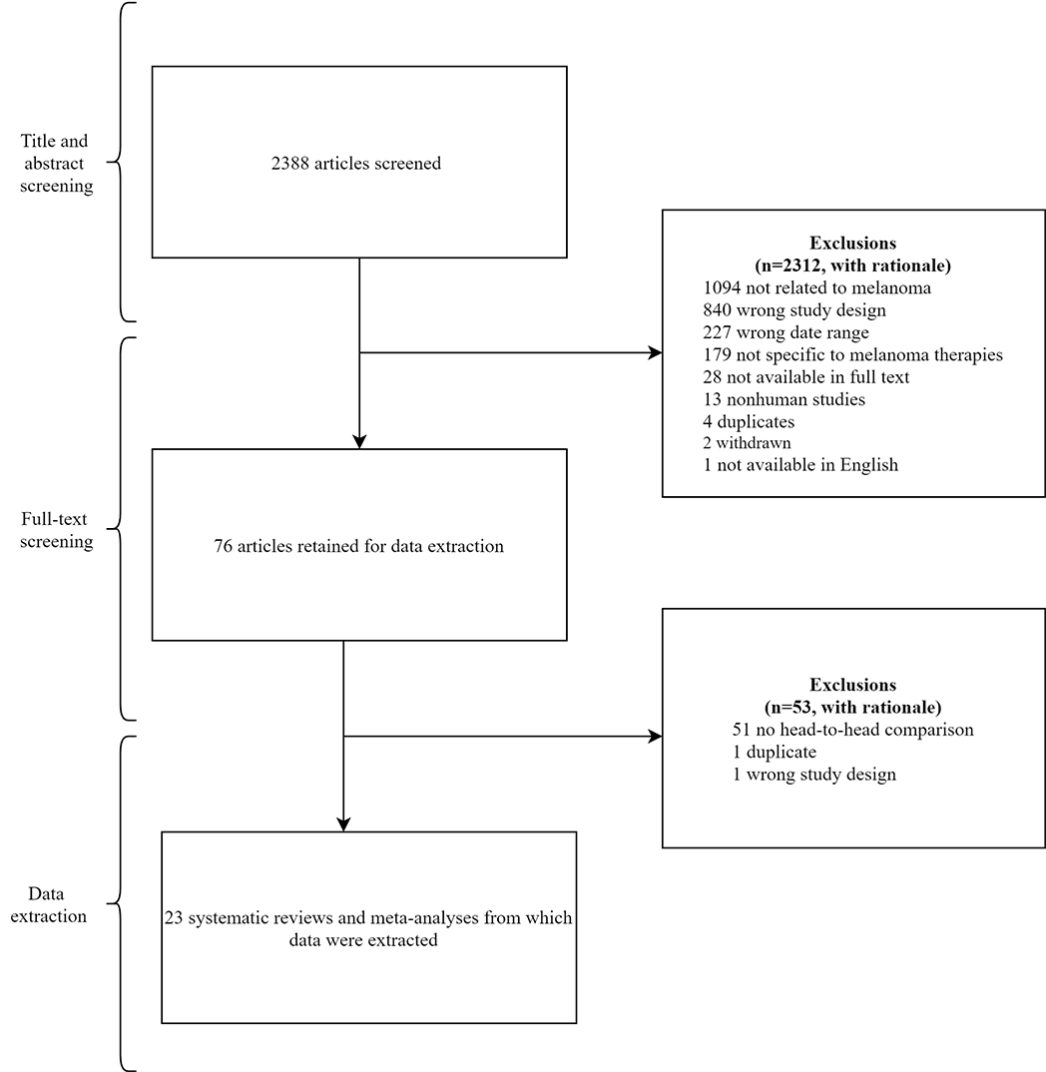
PRISMA (Preferred Reporting Items for Systematic Reviews and Meta-Analyses) flowchart for included studies.

Our search of MEDLINE and Embase yielded 2388 records. A total of 2312 records were excluded after title and abstract screening. Full-text screening led to the exclusion of an additional 53 records. A total of 23 systematic reviews with or without meta-analyses investigating treatment interventions for melanoma were included (Figure 2).

### Systematic Review Characteristics

The 23 systematic reviews included in our final sample were conducted by 120 authors and published within 21 journals. Systematic reviews investigated pharmacologic interventions (8/23, 35%), surgical interventions (7/23, 30%), or a multidisciplinary treatment approach (8/23, 35%). Of the 23 systematic reviews, 19 (83%) reported that none of the authors had a COI, 2 (9%) reported that at least one author had a COI, and 2 (9%) failed to provide a COI disclosure statement. Only 1 systematic review out of 23 (4%) was found to have a high RoB. Additional study characteristics are provided in Table 1.

**Table 1:** Characteristics of systematic reviews.

Characteristic and form response			Value (N=23), n (%)
**Journal**			
	International Immunopharmacology	3 (13)	
	Annals of Oncology	1 (4)	
	Anticancer Research	1 (4)	
	Cancer Medicine	1 (4)	
	Cancers	1 (4)	
	Cutaneous and Ocular Toxicology	1 (4)	
	European Journal of Cancer (Oxford, England: 1990)	1 (4)	
	European Journal of Surgical Oncology	1 (4)	
	Head & Neck	1 (4)	
	American Journal of Rhinology & Allergy	1 (4)	
	JAMA Network Open	1 (4)	
	Journal of Dermatological Treatment	1 (4)	
	Journal of Oncology	1 (4)	
	Journal of the American Academy of Dermatology	1 (4)	
	Journal of the European Academy of Dermatology and Venereology	1 (4)	
	OncoTargets and Therapy	1 (4)	
	Oncotarget	1 (4)	
	Technology in Cancer Research & Treatment	1 (4)	
	The British Journal of Surgery	1 (4)	
	The Journal of Laryngology and Otology	1 (4)	
	The Laryngoscope	1 (4)	
**Accuracy of author conflict of interest (COI) disclosure statement (N=120 authors)**			
	No COI found	95 (79)	
	All COIs completely disclosed in systematic review	20 (17)	
	Incomplete COI disclosure (found to have disclosed and undisclosed)	5 (4)	
**Intervention type**			
	Drug	8 (35)	
	Multiple	8 (35)	
	Surgical technique or intervention	7 (30)	
**Affiliation of first author**			
	Public academic institution	16 (69)	
	Government	4 (17)	
	Private academic institution	3 (13)	
**Affiliation of last author**			
	Public academic institution	17 (74)	
	Government	3 (13)	
	Private academic institution	3 (13)	
**Source of funding**			
	No funding received	8 (35)	
	Public	8 (35)	
	No statement present	6 (26)	
	University	1 (4)	
**COI statement**			
	All authors report no COIs	19 (83)	
	One or more authors report a COI	2 (9)	
	No COI statement present	2 (9)	
**Self-citation of primary studies**			
	Yes, included one or more self-cited primary studies	4 (17)	
	No, did not include self-cited primary studies	19 (83)	

### Author Characteristics and Completeness of COI Disclosures

Of the 120 authors, 25 (20.8%) were found to have a COI, either disclosed, undisclosed, or both. Of these 25 authors, 20 (80%) reported no COI within the review’s disclosure statement but were found to have an undisclosed COI. The remaining 5 authors out of 25 (20%) disclosed one or more COI but were found to have an additional undisclosed COI that was omitted from the COI disclosure statement (Table 1).

### Relationship Between COI and Favorability of Results and Conclusions

Of the 12 systematic reviews with one or more authors with a COI, 7 (58%) reported narrative results favoring the treatment group and 9 (75%) reported conclusions favoring the treatment group. Of the 11 systematic reviews with no conflicted authors, 4 (36%) reported results favoring the treatment group and 5 (46%) reported conclusions favoring the treatment group. Our results showed no statistically significant association between author COIs and the favorability of results (*P*=.53) or conclusions (*P*=.15) (Table 2).

**Table 2:** Association between favorability of results and conclusions, risk of bias, and conflicts of interest (COIs) among systematic review authors.

Review outcomes			COIs among systematic review authors, n (%)				*P* value^a^
			No COIs (n=11)		COIs (n=12)		
**Favorability of results**							.53
	Results favor treatment group	4 (36)		7 (58)			
	Results are mixed or inconclusive	1 (9)		0 (0)			
	Results favor placebo or control group	6 (55)		5 (42)			
**Favorability of discussion and conclusions**							.15
	Discussion favors treatment group	5 (45)		9 (75)			
	Discussion is mixed or inconclusive	0 (0)		0 (0)			
	Discussion favors placebo or control group	6 (55)		3 (25)			
Risk of bias: high			0 (0)		1 (8)		.52

^a^*P* values were calculated from Fisher exact tests.

### Relationship Between Sponsorship and Favorability of Results and Conclusions

Of the 23 systematic reviews, 9 (39%) received funding support, 8 (35%) did not receive funding support, and 6 (26%) did not provide a funding statement. Of the 9 reviews receiving nonindustry support, 3 (33%) reported results favoring the treatment group and 3 (33%) reported conclusions favoring the treatment group (Table 3). Because our sample did not include a single industry-funded systematic review, we could not assess for a relationship between industry sponsorship and the favorability of review results and conclusions.

**Table 3:** Association between favorability of results and conclusions, risk of bias, and systematic review sponsorship.

Review outcomes			Funding details, n (%)						
			Industry (n=0)		Nonindustry (n=9)		No funding received (n=8)		No statement listed (n=6)
**Favorability of results**									
	Results favor treatment group	0 (0)		6 (67)		3 (38)		2 (33)	
	Results are mixed or inconclusive	0 (0)		0 (0)		0 (0)		1 (17)	
	Results favor placebo or control group	0 (0)		3 (33)		5 (62)		3 (50)	
**Favorability of discussion and conclusions**									
	Discussion favors treatment group	0 (0)		8 (88)		3 (37)		3 (50)	
	Discussion is mixed or inconclusive	0 (0)		0 (0)		0 (0)		0 (0)	
	Discussion favors placebo or control group	0 (0)		1 (11)		5 (62)		3 (50)	
**Risk of bias**									
	High risk of bias	0 (0)		1 (11)		0 (0)		0 (0)	
	Low risk of bias	0 (0)		8 (89)		8 (100)		6 (100)	

### Relationship Between RoB, Industry Sponsorship, and COIs

Only 1 of the 23 systematic reviews (4%) was found to have a high RoB. This systematic review received nonindustry support and was conducted by one or more authors with at least one COI. Because only 1 systematic review had a high RoB, we were unable to determine whether high RoB influenced the nature of review results and conclusions.

## Discussion

### Principal Findings

The results of our study indicate that COIs are a regular, often incompletely disclosed, occurrence in systematic reviews investigating melanoma interventions. Roughly one-half of the included systematic reviews were authored by at least one author with a potential COI. Additionally, one-fifth of the systematic review authors did not fully disclose all potential COIs. Previous work using the CMS Open Payments database to detail physician-industry relationships found variable rates of undisclosed COIs among clinical practice guidelines authors in multiple disciplines [30-33]. For example, undisclosed COIs were found to be present for 45% (22/49) of authors in dermatology [30], 6% (3/49) of authors in otolaryngology [31], 31% (23/74) of authors in orthopedics [32], and 20% (20/54) of authors in urology [33]. Similarly, of the 9 authors from our sample who were found on the CMS Open Payments database, all had at least one undisclosed COI that was omitted from the systematic review COI disclosure statement. In an assessment of the association between COIs and results, conclusions, and methodological quality, Hansen et al [34] found that systematic reviews with a COI were more likely to have favorable conclusions than those without a COI. Although our analysis failed to identify a similar association between author COIs and review outcomes, the high rates of undisclosed COIs among authors included in our sample highlight the need for more complete COI disclosure. Inconsistency in the completeness of COI disclosure is evident, and one potential explanation may be a lack of adherence to a comprehensive, more uniform disclosure guideline.

Complete disclosure of COIs is a widespread issue, and the lack of standardization of disclosure requirements between journals could partially explain the high rate of undisclosed COIs in our sample. For example, a study by Zhu and Sun [35] found that only 31% of medical journals mentioned a COI policy, 7% required a COI statement, and 4% standardized the COI submissions form. In addition to the inconsistent presence of journal COI disclosure policies, journals often fail to clearly outline expectations regarding COI disclosure requirements, making it difficult or impossible to establish what COI information should be disclosed. For instance, a 2007 study by Ancker and Flanagin [36] determined that only 68% of journals provided examples of what may be perceived as a potential COI and only 46% of journals explicitly defined the term. The same authors reported, upon initial attempts, that they were only able to locate COI disclosure policies for 33% of “high-impact, peer-reviewed” journals. Results from studies such as these highlight that, even when COI disclosure policies are present, authors are often left to determine for themselves what information should be disclosed at the time of manuscript submission.

Resnik and Elliott [37] reached a similar conclusion concerning the potential influence of financial biases on the design and interpretation of the study. These authors highlighted the difficulties in judging a study on scientific merit alone and presented methods to take financial relationships into account, without crudely discrediting the results of the study. To help address financial bias in medical literature, we recommend an initiative to develop a similar database to the CMS Open Payments database that would centralize potential COIs, financial and otherwise, and include all stakeholders in academic medicine (eg, clinicians, researchers, editors, funders, and peer reviewers), as well as research stakeholders with non-US–based affiliations.

Furthermore, we encourage readers to consider COIs when interpreting the results of systematic reviews. Analyses designed to define the prevalence of undisclosed and disclosed COIs in medical literature may increase awareness and emphasis on the issues surrounding COIs and lead to more standardized disclosure policies, such as an ICMJE COI form expanded to a global scale [20]. Perhaps a more comprehensive and enforced implementation of a standardized COI form would decrease the chances of potential COIs remaining undisclosed. Even though complete COI disclosure may be a step in the right direction, it can be difficult to interpret the degree of influence these COIs have on the procedures and results of a study. Maharaj [38] attempted to solve this issue by developing a COI scale that provides a numerical score for a study based on the potential bias risk from the disclosed COIs. Scales similar to that of Maharaj could be used as a means to compare the degree of influence that disclosed COIs have in a systematic review [38]. These measures may aid in improving transparency and accessibility in medical research, dermatological and otherwise.

Our study had several strengths. The design of our analysis maximized our ability to locate and confirm potential undisclosed COIs. Our protocol was established a priori and was published for reference on Open Science Framework with other materials and protocol amendments to increase transparency and reproducibility. Data extraction was carried out as recommended by the Cochrane Handbook [39]. Prior to the study, investigators received training to account for any differences in investigational analysis, data extraction was standardized using pilot-tested Google Forms, and a search string–generating program was used to promote search uniformity. Limitations of our study include a small sample size and difficulty verifying authors with common names on patent websites. In addition, our sample lacked industry-sponsored systematic reviews, thereby preventing further analysis into the role that industry may have on the nature of reported results and conclusions. Taken together, COIs and industry sponsorship may affect the favorability of study outcomes, but the source of the discrepancy in favorability between systematic reviews with COIs and those without remains unclear [8,30,40].

### Conclusions

COIs are common, yet often incompletely disclosed, in systematic reviews investigating melanoma interventions. However, our results suggest that the presence of author COIs did not influence the favorability of reported outcomes of melanoma systematic reviews. Future investigations are needed to more fully evaluate the influence that COIs and industry sponsorship may have on the nature and direction of results and conclusions within published dermatology literature.

## Appendix

Multimedia Appendix 1 MEDLINE and Embase searches for systematic reviews and meta-analyses regarding melanoma interventions.

Multimedia Appendix 2 Description of databases used to search for undisclosed conflicts of interest among systematic review authors.

## Abbreviations

CMS: Centers for Medicare & Medicaid Services
COI: conflict of interest
DOI: Digital Object Identifier
ICMJE: International Committee of Medical Journal Editors
PRISMA: Preferred Reporting Items for Systematic Reviews and Meta-Analyses
PRISMA-P: Preferred Reporting Items for Systematic Reviews and Meta-Analyses for Protocols
RoB: risk of bias
USPTO: United States Patent and Trademark Office
