# Supplementary material for: Conflicts of Interest Among Authors of Systematic Reviews and Meta-analyses Investigating Interventions for Melanoma: Cross-sectional Literature Study
Source: JMIR Dermatol. 2021 Jun 7;4(1):e25858. doi: 10.2196/25858 (PMC10501528; doi:10.2196/25858)
Supplement: Multimedia Appendix 1 [file derma_v4i1e25858_app1.docx]

| **Ovid MEDLINE:**  1. exp Melanoma/  2. (melanoma* or (pigment* adj1 cancer*) or melanocarcinoma* or nevocarcinoma*).mp.  3. 1 or 2  4. exp Therapeutics/  5. (treat* or therap* or help* or interven*).mp.  6. 4 or 5  7. 3 and 6  8. exp Melanoma/dh, dt, th [Diet Therapy, Drug Therapy, Therapy]  9. 7 or 8  10. exp "Systematic Review"/  11. exp Meta-Analysis/  12. ("systematic review" or "meta-analysis" or (systematic* adj1 review*)).ti,ab.  13. 10 or 11 or 12  14. 9 and 13 | **Ovid Embase:**  1. exp melanoma/  2. (melanoma* or (pigment* adj1 cancer*) or melanocarcinoma* or nevocarcinoma*).mp.  3. 1 or 2  4. exp therapy/  5. (treat* or therap* or help* or interven*).mp.  6. 4 or 5  7. 3 and 6  8. exp melanoma/dm, dt, th [Disease Management, Drug Therapy, Therapy]  9. 7 or 8  10. exp "systematic review"/  11. exp meta analysis/  12. ("systematic review" or "meta-analysis" or (systematic* adj1 review*)).ti,ab.  13. 10 or 11 or 12  14. 9 and 13 |
| --- | --- |
