# Supplementary material for: Conflicts of Interest Among Authors of Systematic Reviews and Meta-analyses Investigating Interventions for Melanoma: Cross-sectional Literature Study
Source: JMIR Dermatol. 2021 Jun 7;4(1):e25858. doi: 10.2196/25858 (PMC10501528; doi:10.2196/25858)
Supplement: Multimedia Appendix 2 [file derma_v4i1e25858_app2.docx]

| **Database** | **Description of Database** |
| --- | --- |
| Open Payments Database  (https://openpaymentsdata.cms.gov) | Open Payments Database – which was created on September 1, 2013 – is a Congressionally-mandated, openly accessible resource designed to increase the transparency within the United States healthcare system. This database collects and publishes information regarding industry relationships between healthcare providers (eg, physicians and teaching hospitals) and industry (eg, drug and device manufacturers). Physicians and teaching hospitals report industry payments received in the form of research, food and beverage, travel, and consulting or speaking fees. |
| Pro Publicas Dollars for Profs  (https://projects.propublica.org/dollars-for-profs) | Dollars for Profs provides information from state universities and the National Institutes of Health (NIH) regarding industry payments and conflicts of interest of academic professors, researchers, and other support personnel. Rationale for including this database was based on the knowledge that searching for non-healthcare professionals listed as an author of a systematic review included in our sample would not return beneficial information. |
| United States Patent and Trademark Office (USPTO)  (https://www.uspto.gov) | The United States Patent and Trademark Office (USPTO) is responsible for the registration of US patents and trademarks in accordance with the commerce clause (Article I, Section 8, Clause 3) of the US Constitution. In addition, the USPTO “furthers effective intellectual property protection for U.S. innovators and entrepreneurs worldwide by working with other agencies to secure strong IP provisions in free trade and other international agreements.” (https://www.uspto.gov/about-us) |
| Google Patents  (https://patents.google.com) | Google Patents is a database consisting of greater than 120 million patent publications from more than 100 different patent offices worldwide. In addition, Google Patents provides access to technical documents and books indexed in Google Scholar and Google Books, as well as documents included in the Prior Art Archive. |
| PubMed  (https://pubmed.ncbi.nlm.nih.gov) | PubMed was launched in January 1996 and is one of the most widely used databases for academic research. The entire MEDLINE collection includes more than 30 million citations from biomedical literature. As part of the Enterz system of informational retrieval, PubMed is maintained by The United States National Library of Medicine at the National Institutes of Health. (https://pubmed.ncbi.nlm.nih.gov/) |
